# Nutritional approaches to slow late finishing pig growth: implications on carcass composition and pork quality

**DOI:** 10.1093/jas/skaa368

**Published:** 2020-12-05

**Authors:** Emma T Helm, Jason W Ross, John F Patience, Steven M Lonergan, Elisabeth Huff-Lonergan, Laura L Greiner, Leah M Reever, Chad W Hastad, Emily K Arkfeld, Nicholas K Gabler

**Affiliations:** 1 Department of Animal Science, Iowa State University, Ames, IA; 2 Iowa Pork Industry Center, Iowa State University, Ames, IA; 3 Department of Food Science and Human Nutrition, Iowa State University, Ames, IA; 4 New Fashion Pork, Inc., Jackson, MN; 5 Triumph Foods, St. Joseph, MO

**Keywords:** carcass composition, fresh pork quality, growth restriction, nutrition, social vices, swine

## Abstract

Although pork producers typically aim to optimize growth rates, occasionally it is necessary to slow growth, such as when harvest facility capacity is limited. In finishing pigs, numerous dietary strategies can be used to slow growth so pigs are at optimal slaughter body weights when harvest facility capacity and/or access is restored. However, the impact of these diets on pork carcass quality is largely unknown. Thus, this study aimed to evaluate the efficacy of dietary strategies to slow growth in late finishing pigs and evaluate their effects on carcass composition and pork quality. Mixed-sex pigs (*n* = 897; 125 ± 2 kg BW) were randomly allotted across 48 pens and assigned to 1 of 6 dietary treatments (*n* = 8 pens/treatment): (1) Control diet representative of a typical finisher diet (**CON**); (2) diet containing 3% calcium chloride (**CaCl**_**2**_); (3) diet containing 97% corn and no soybean meal (**Corn**); (4) diet deficient in isoleucine (**LowIle**); (5) diet containing 15% neutral detergent fiber (**NDF**) from soybean hulls (15% NDF); and (6) diet containing 20% NDF from soybean hulls (20% NDF). Over 42 d, pen body weights and feed disappearance were collected. Pigs were harvested in 3 groups (14, 28, and 42 d on feed) and carcass data collected. From the harvest group, 1 loin was collected from 120 randomly selected carcasses (20 loins/treatment) to evaluate pork quality traits. Overall, ADG was reduced in CaCl_2_, Corn, and 20% NDF pigs compared with CON pigs (*P* < 0.001). However, ADFI was only reduced in CaCl_2_ and 20% NDF pigs compared with CON (*P* < 0.001). Feed efficiency was reduced in CaCl_2_ and Corn pigs compared with CON (*P* < 0.001). Hot carcass weights were reduced in CaCl_2_ pigs at all harvest dates (*P* < 0.001) and were reduced in Corn and 20% NDF pigs at days 28 and 42 compared with CON pigs (*P* < 0.001). In general, CaCl_2_ and 20% NDF diets resulted in leaner carcasses, whereas the Corn diet increased backfat by 42 d on test (*P* < 0.05). Loin pH was reduced and star probe increased in CaCl_2_ pigs compared with CON pigs (*P* < 0.05); no treatments differed from CON pigs regarding drip loss, cook loss, color, firmness, or marbling (*P* ≥ 0.117). Overall, these data indicate that several dietary strategies can slow finishing pig growth without evidence of behavioral vices. However, changes to carcass composition and quality were also observed, indicating quality should be taken into consideration when choosing diets to slow growth.

## Introduction

The overarching goal of any swine production system is to efficiently optimize growth rates and/or feed intake to enhance barn throughput, lean tissue accretion, and profitability. Occasionally circumstances arise in which it may be necessary to slow growth rates. Although rare, these instances could include interruptions in feed supply, animal movement restrictions as a result of a disease outbreak, or when harvest facility access is suspended or delayed (Weng et al., 2017). Most recently, supply chain disruptions were observed in the United States during the spring of 2020, with the shuttering of numerous pork harvest facilities across the country due to the COVID-19 pandemic. On April 29, 2020, the pork industry observed the lowest harvest facility capacity utilization of the year, with only 53.9% of harvest facility capacity being operational (Hahn et al., 2020). Although harvest facility capacity did rebound quickly after harvest facilities began to reopen, complications arising from having so many simultaneous harvest facility closures revealed critical areas in the hog production supply chain that suffer during harvest facility disruptions. One of these was the ability to slow the growth of late finisher, market-ready hogs until harvest facilities became available.

Besides mass de-population, dietary manipulation of pig feed intake and growth can be a viable option for pork producers to slow or stop pig growth. Numerous dietary strategies can be implemented to slow growth and/or reduce feed intake in finishing pigs. These include adding acidogenic salts such as anhydrous calcium chloride to suppress appetite, reducing essential amino acids to restrict lean growth, and increasing neutral detergent fiber (**NDF**) content to lower dietary energy density to reduce feed intake. However, a paucity of published data are available evaluating these dietary strategies to slow or stop pig growth. Further, there is concern that feeding diets intended to slow growth rates may increase behavioral vices such as ear and tail biting, as caloric restriction (via feed restriction) in finishing pigs is known to increase aggression (Hessel et al., 2006). However, if this increased aggression occurs in pigs fed growth limiting, yet ad libitum fed, diets is unknown. There is also concern that these diets could have unintended consequences on pork carcass composition or pork quality. For instance, feeding a high corn, protein deficient diet may increase carcass backfat (Kerr et al., 1995). In addition, acidogenic diets could increase the incidence of pale, soft, exudative (**PSE**) pork (Ahn et al., 1992; Moore et al., 2016).

An understanding of which dietary strategies work to slow growth of late finishing pigs is necessary to prepare for future market disruptions. Beyond describing growth characteristics, it is also necessary to characterize how these dietary strategies affect the final fresh pork product. Thus, the objective of this study was to evaluate multiple diets designed to slow growth in commercially housed late finishing pigs and to evaluate the effect of these dietary strategies on pig behavioral vices, carcass composition, and fresh pork loin quality.

## Materials and Methods

All animal procedures were approved by the Iowa State University Institutional Animal Care and Use Committee (IACUC protocol #20-057) and adhered to the ethical and humane use of animals for research. All live animal research was conducted at the New Fashion Pork Freking R 071 Finisher Research site, Round Lake, MN, between May and June of 2020. The barn consisted of a tunnel ventilation design, with each pen providing 0.74 m^2^ of space per pig when stocked with 19 pigs/pen. Each pen had 1 water cup per pan per pen and a 3-hole feeder, wherein each hole was 40.6 cm wide and provided 6.35 cm of feeder spacer per pig.

### Animals, housing, and dietary treatments

A total of 897 late finishing mixed sex pigs (125 ± 2 kg BW; Fast LW × PIC LR × TR437) free of clinical or subclinical pathogenic disease were randomly selected, allocated across 48 pens, with 18 (15 pens) or 19 (33 pens) pigs housed per pen. Pens were blocked by initial inventory number and assigned to 1 of 6 dietary treatments (*n* = 8 pens/treatment) as follows: (1) Control diet representative of a typical corn–soybean meal-based finisher diet (**CON**); (2) diet containing 3% anhydrous calcium chloride (**CaCl**_**2**_); (3) diet containing 97% corn and no soybean meal or synthetic amino acids (**Corn**); (4) diet formulated as per CON but deficient in isoleucine (**LowIle**); (5) diet containing 15% NDF from soybean hulls (**15% NDF**); and (6) diet containing 20% NDF from soybean hulls (**20% NDF**). Diet formulation and calculated nutrient composition is presented in Table 1. Regardless of dietary treatment, pigs were ad libitum fed and had free access to water at all times. Pen body weights and feed disappearance were recorded at days 0, 14, 28, and 42 d to calculate average daily gain (**ADG**), average daily feed intake (**ADFI**), and feed efficiency (gain:feed, **G:F**).

**Table 1.** Diet formulation and calculated nutrient composition, as fed

Ingredient	CON	CaCl_2_	Corn^1^	LowIle	15% NDF	20% NDF
Corn	81.03	78.72	97.21	85.97	69.79	51.62
Soybean meal, 46.5% CP	16.05	16.25	—	10.60	14.90	13.20
Limestone	1.48	—	1.58	1.43	1.25	0.98
Beef tallow	0.50	0.50	0.50	0.50	0.50	0.50
Salt	0.50	0.50	0.53	0.51	0.50	0.51
l-Lysine HCl	0.25	0.25	—	0.41	0.25	0.25
l-Threonine	0.06	0.06	—	0.13	0.06	0.08
dl-Methionine	0.01	0.02	—	0.05	0.03	0.06
l-tryptophan	0.01	0.01	—	0.04	0.01	0.02
l-Valine	—	—	—	0.09	—	—
Monocalcium phosphate, 21%	—	0.58	0.08	0.18	0.10	0.18
Vitamin/trace mineral premix^2^	0.10	0.10	0.10	0.10	0.10	0.10
Phytase^3^	0.01	0.01	0.01	0.01	0.01	0.01
Soybean hulls	—	—	—	—	12.50	32.50
Calcium chloride, anhydrous	—	3.00	—	—	—	—
Calculated composition						
Dry Matter, %	84.2	84.5	83.8	84.1	85.0	86.3
ME, Mcal/kg	3.28	3.21	3.32	3.30	3.11	2.83
NE, Mcal/kg	2.50	2.44	2.56	2.52	2.30	2.00
Crude protein, %	12.6	12.5	6.0	10.8	12.6	12.8
Crude fat, %	3.17	3.11	3.42	3.24	2.98	2.66
Crude fiber, %	1.88	1.85	1.59	1.77	6.12	12.92
Acid detergent fiber, %	2.71	2.67	2.12	2.50	7.59	15.40
NDF, %	6.80	6.64	6.99	6.82	13.34	23.81
Total calcium, %	0.66	1.01	0.65	0.65	0.65	0.65
Total phosphorus, %	0.33	0.45	0.29	0.35	0.33	0.31
Available phosphorus, %^4^	0.21	0.31	0.20	0.23	0.22	0.20
SID lysine, %	0.74	0.74	0.16	0.73	0.74	0.74
Lys:ME,g/Mcal	2.24	2.30	0.47	2.22	2.37	2.61
Calculated SID AA:Lys						
SAA:Lys	0.51	0.51	1.44	0.50	0.50	0.48
Thr:Lys	0.61	0.60	1.12	0.60	0.60	0.61
Trp:Lys	0.18	0.18	0.26	0.18	0.17	0.18
Val:Lys	0.66	0.66	1.49	0.66	0.65	0.63
Ile:Lys	0.57	0.57	1.02	0.45	0.56	0.54
Leu:Lys	1.32	1.31	3.80	1.15	1.27	1.18

^1^Diet included corn plus vitamins, trace minerals and macrominerals to meet the pigs’ NRC requirements for these nutrients.

^2^The vitamin and trace mineral premix provided per kilogram of complete diet: 5,526 IU vitamin A, 250 IU vitamin D3, 17.5 μg vitamin D, 27 IU vitamin E, 22 μg vitamin B12, 4.5 mg riboflavin, 25.5 mg niacin, 16.4 mg d-pantothenic acid, 2.3 mg menadione, 33 ppm Cu as copper Intellibond, 81 ppm Fe as ferrous sulfate, 0.4 ppm I as ethylenediamine dihydroiodide and calcium iodate, 23 ppm Mn as Availa-Mn, 0.66 ppm Se as sodium selenite, and 198 ppm Zn as zinc Intellibond.

^3^Quantum Blue 10P, Ab Vista Feed Ingredients; Marlborough, Wiltshire, UK. Minimum activity of 5,000 FTU/g product.

^4^Includes 0.15% phosphorus released from phytase.

### Assessment of behavioral vices

Pigs were evaluated by the same individual blinded to treatment groups at days 7, 14, and 21 for the number of pigs with scratches/sores, number of pigs appearing pale or anemic, and the number of pigs appearing dehydrated within each pen. Dehydration was classified with pigs having sunken eyes, tacky, or dry oral mucous membranes and/or gaunt appearance. Ear lesions were scored on a scale of 0 to 2, with 0 = no visible lesions; 1 = skin lesion affecting ≤¼ area of the ear edges or surface; and 2 = skin lesion affecting >¼ area of the ear edges or surface. Tail biting was scored on a scale of 0 to 4, with 0 = no visible lesions; 1 = superficial lesions, points, and lines; 2 = deeper skin lesion of small size (maximum size equal to tail diameter at respective location); 3 = deeper skin lesion of larger size (larger than tail diameter at respective location); and 4 = loss of tail length due to biting.

### Carcass composition and pork quality assessment

At days 14, 28, and 42, an equivalent number of pigs per pen were selected for harvest based on the highest body weight estimates (day 14 = 3 pigs/pen; day 28 = 6 pigs/pen; day 42 = 10 pigs/pen). Prior to harvest, pigs were individually tagged and tattooed. Pigs were shipped to a commercial harvest facility (Triumph Foods, St. Joseph, MO) for collection of carcass composition data (backfat depth, loin depth, and percent lean) following methodology outlined by Lerner et al. (2020).

At day 28, 120 pigs (8 pens/treatment; 20 loins/treatment) were randomly selected for further pork quality assessment. One loin from each pig was collected and shipped to the Iowa State University Meat Laboratory (Ames, IA) and stored at 2 °C for 7 d postmortem in vacuum-sealed bags prior to analysis. Boneless loins were then weighed and separated into chops for analysis. Ultimate pH was recorded using a calibrated Hanna HI9025 pH meter (Hanna Instruments, Woonsocket, RI) with a penetration probe. Lean tissue color was determined by Hunter L, a, and b color values (L: lightness, a: redness, and b: yellowness) using a calibrated Konica Minolta colorimeter (Konica Minolta Sensing Americas Inc., Ramsey, NJ) with a D65 light source, 50 mm aperture, and 2° observer angle. Marbling, color, and firmness scores were assigned using standards established by the National Pork Board (NPPC, 2000). Marbling was scored on a 10-point scale with 1 = 1.0% intramuscular fat and 10 = 10.0% intramuscular fat. Loin color was scored on a 6-point scale with 1 = pale pinkish-grey to white and 6 = dark purplish red. Firmness was scored on a 3-point scale with 1 = very soft and 3 = very firm.

Drip loss was determined on 2 chops (2.5 cm thick) per loin (Lonergan et al., 2001). Briefly, chops were trimmed of external fat, weighed, and stored for 24 hr in a sealed plastic bag at 4 °C. After storage, the liquid lost was removed from each bag, and the chops were blotted of excess moisture and reweighed. Drip loss was recorded as a percentage of the original weight of the chop by the following equation: [(initial weight – final weight)/initial weight] × 100.

One 6 cm thick section from each loin was vacuum sealed, frozen, and stored (‒20 °C). Samples were thawed overnight at 4 °C, after which one 2.5 cm chop was cut, trimmed of external connective and adipose tissue, weighed, and cooked to an internal temperature of 63 °C using a Ninja Grill (Model AG300, SharkNinja, Needham, MA). Cook loss was determined using the post cooking weight with the following formula: [(raw chop weight – cooked chop weight)/raw chop weight] × 100. Star probe was measured on 1 cooked chop per loin using an Instron (Instron, Norwood, MA) fitted with a 5-point star probe attachment. Three compressions (20% of original height) were made on each chop and averaged for a final star probe value in peak force (Schulte et al., 2019).

### Statistical analysis

Statistical analysis of all data was performed using SAS (version 9.4; SAS Inst. Inc., Cary, NC). The following mixed model was fitted to growth performance parameters with pen as the experimental unit:

$$\begin{array}{l} Y_{ijk}=\mu +Diet_{i}+Block_{j}+e_{ijk} \end{array}$$

wherein *Y*_ijk_ = the phenotype measured on pen *k*; μ = the overall mean; Diet_i_ = effect of dietary treatment (fixed effect); Block_j_ = blocking factor of initial inventory (random effect); and e_ijkl_ = error term of pen *k* subjected to treatment *i* in block *j*, e_ijk_ ~ N(0, σ _e_^2^). Carcass composition and pork loin quality data were assessed using a similar mixed model as above, except pig was used as the observational unit within the experimental unit of pen (*n* = 8 pens/treatment), and the random effect of pen within block and treatment was included to account for this subsampling. Least squares means were determined using the LS means statement and differences in Least squares means were produced using the pdiff option using the Tukey’s adjustment to control for multiple comparisons. All data are presented as Least squares means with a pooled standard error. Differences were considered significant when *P* < 0.05 and a tendency when 0.05 ≤ *P* ≤ 0.10.

## Results

### Growth performance and behavioral vices

Initial body weight (125 kg) did not differ among treatments (*P* = 1.000; Table 2). In the first 14 d on test diets, ADG tended to be reduced in pigs fed the 20% NDF diet (Table 2; *P* = 0.066) compared with pigs fed the CON diet and was reduced in pigs fed both Corn (41%; *P* < 0.001) and CaCl_2_ (240%; *P* < 0.001) diets compared with the CON diet. However, ADFI in the first 14 d was only reduced in pigs fed the 20% NDF (21%; *P* < 0.001) and CaCl_2_ (60%; *P* < 0.001) diet compared with the CON diet. As such, G:F was reduced in pigs fed both Corn (42%; *P* = 0.001) and CaCl_2_ (226%; *P* < 0.001) diets compared with the CON diet. When the first group of pigs was marketed at day 14, average pig BW tended to be reduced in pigs fed the 20% NDF diet (*P* = 0.075) compared with the CON diet and were significantly reduced in pigs fed the Corn (4%; *P* < 0.001) and CaCl_2_ (14%; *P* < 0.001) diets compared with the CON diet.

**Table 2.** Growth performance of pigs fed diets intended to slow growth

Item	CON	CaCl_2_	Corn	LowIle	15% NDF	20% NDF	SEM	*P*-value
Day 0 BW, kg^1^	125.0	125.0	125.0	125.0	125.0	125.0	1.67	1.000
Days 0 to 14^1^								
ADG, kg	0.95^ab^	−0.41^d^	0.57^c^	0.90^ab^	1.02^a^	0.83^b^	0.031	<0.001
ADFI, kg	3.01^a^	1.12^c^	3.14^a^	2.99^a^	2.98^a^	2.38^b^	0.051	<0.001
G:F	0.32^a^	−0.38^c^	0.18^b^	0.30^a^	0.34^a^	0.35^a^	0.023	<0.001
Day 14 BW, kg^1^	138.4^ab^	119.3^d^	133.0^c^	137.7^ab^	139.3^a^	136.6^b^	1.64	<0.001
Days 15 to 28^2^								
ADG, kg	0.64^a^	0.40^b^	0.36^b^	0.60^a^	0.59^a^	0.54^ab^	0.043	<0.001
ADFI, kg	3.11^a^	2.09^b^	3.04^a^	2.92^a^	3.22^a^	2.88^a^	0.107	<0.001
G:F	0.21^a^	0.19^a^	0.12^b^	0.20^a^	0.18^a^	0.19^a^	0.010	<0.001
Day 28 BW, kg^2^	147.3^a^	124.9^d^	138.0^c^	146.1^ab^	147.6^a^	144.1^b^	1.52	<0.001
Days 29 to 42^3^								
ADG, kg	0.60^a^	0.15^c^	0.25^bc^	0.48^ab^	0.62^a^	0.41^ab^	0.066	<0.001
ADFI, kg	3.02^ab^	2.14^c^	2.93^ab^	2.91^b^	3.18^a^	2.88^b^	0.063	<0.001
G:F	0.20^a^	0.07^c^	0.09^bc^	0.16^ab^	0.19^a^	0.14^abc^	0.022	<0.001
Day 42 BW, kg^3^	155.7^a^	127.0^d^	141.5^c^	152.8^ab^	156.2^a^	149.8^b^	1.95	<0.001
Overall (days 0 to 42)^1, 2, 3^								
ADG, kg	0.73^a^	0.05^d^	0.39^c^	0.66^ab^	0.74^a^	0.59^b^	0.022	<0.001
ADFI, kg	3.05^a^	1.78^c^	3.04^a^	2.94^ab^	3.13^a^	2.71^b^	0.055	<0.001
G:F	0.24^a^	0.03^c^	0.13^b^	0.23^a^	0.24^a^	0.22^a^	0.007	<0.001

^1^ *n* = 8 pen/trt, 19 pigs/pen.

^2^ *n* = 8 pen/trt, 16 pigs/pen.

^3^ *n* = 8 pen/trt, 10 pigs/pen.

^a–d^Means within a row with differing superscripts differ significantly at *P* < 0.05.

Days 15 to 28 provided similar growth performance observations with ADG being reduced in pigs fed both Corn (44%; *P* = 0.001) and CaCl_2_ (38%; *P* = 0.004) diets compared with the CON diet (Table 2). Average daily feed intake was only reduced in pigs fed the CaCl_2_ diet (33%; *P* < 0.001) compared with the CON diet. Feed efficiency was reduced in pigs fed the Corn diet (42%; *P* < 0.001) compared with the CON diet. At day 28, when the second group of pigs was marketed, average BW was reduced in pigs fed 20% NDF (2%; *P* = 0.046), Corn (6%; *P* < 0.001), and CaCl_2_ (15%; *P* < 0.001) diets compared with CON, which averaged 147 kg BW.

From days 29 to 42, ADG was reduced in pigs fed Corn (58%; *P* = 0.001) and CaCl_2_ (74%; *P* < 0.001) diets compared with CON (Table 2). Average daily feed intake was reduced in pigs fed the CaCl_2_ (29%; *P* < 0.001) diet compared with CON and G:F was reduced in pigs fed the Corn (57%; *P* = 0.003) diet compared with CON. At day 42, average BW of the final group of pigs harvested was reduced in pigs fed 20% NDF (4%; *P* < 0.001), Corn (9%; *P* < 0.001), and CaCl_2_ (18%; *P* < 0.001) diets compared with CON, which averaged 156 kg.

Overall (days 0 to 42), ADG was reduced in pigs fed 20% NDF (19%; *P* < 0.001), Corn (46%; *P* < 0.001), and CaCl_2_ (94%; *P* < 0.001) diets compared with CON (Table 2). The overall ADFI was only reduced in pigs fed 20% NDF (11%; *P* = 0.002) and CaCl_2_ (42%; *P* < 0.001) diets compared with CON. As such, overall G:F was reduced pigs fed Corn (46%; *P* < 0.001) and CaCl_2_ (89%; *P* < 0.001) diets compared with CON.

Behavioral vices were assessed for the first 21 d of the study (Table 3). Minimal evidence of behavioral vices was observed; thus data were not compared statistically. In total, there were 15 mortalities or removals throughout the 42 d study, which were not increased in diets intended to slow growth. Of the mortalities or removals, 4 were fed the CON diet, 3 fed the CaCl_2_ diet, 2 fed the Corn diet, 1 fed the LowIle diet, 3 fed the 15% NDF diet, and 2 fed the 20% NDF diet. There was an increase in the percentage of pigs that appeared to be dehydrated in each pen, primarily in pigs fed the CaCl_2_ diet. This was unsurprising, given the altered electrolyte balance of this diet and the extremely low feed intake of these pigs, which likely exacerbated their dehydrated appearance. However, there was not a detectable increase in the incidence of ear lesions or tail biting in any dietary treatment group.

**Table 3.** Behavioral vices assessed at days 7, 14, and 21 in pigs fed diets intended to slow growth

Item	CON	CaCl_2_	Corn	LowIle	15% NDF	20% NDF
Day 7						
% pigs in each pen with scratches/sores	0.0	0.0	0.0	0.0	0.0	0.0
% pale/anemic pigs in each pen	0.0	0.0	0.0	0.0	0.0	0.0
% pigs appearing dehydrated in each pen	0.0	97.4	0.0	0.0	0.0	0.0
Ear lesion score^1^	0.0	0.12	0.0	0.0	0.0	0.0
Tail biting score^2^	0.4	0.0	0.0	0.0	0.3	0.3
Day 14						
% pigs in each pen with scratches/sores	0.0	0.0	0.0	0.0	0.0	0.0
% pale/anemic pigs in each pen	0.0	0.0	0.0	0.0	0.0	0.0
% pigs appearing dehydrated in each pen	0.0	97.4	0.0	0.0	0.0	0.0
Ear lesion score^1^	0.0	0.0	0.0	0.0	0.0	0.0
Tail biting score^2^	0.1	0.1	0.0	0.0	0.3	0.3
Day 21						
% pigs in each pen with scratches/sores	0.0	0.0	0.0	0.0	0.0	0.0
% pale/anemic pigs in each pen	0.0	0.0	0.0	0.0	0.0	0.0
% pigs appearing dehydrated in each pen	0.0	100.0	0.0	0.0	0.0	0.0
Ear lesion score^1^	0.0	0.1	0.0	0.1	0.0	0.0
Tail biting score^2^	0.3	0.0	0.0	0.0	0.1	0.0

^1^Scale of 0 to 2, with 0 = no visible lesions; 1 = skin lesion affecting ≤¼ area of the ear edges or surface; and 2 = skin lesion affecting >¼ area of the ear edges or surface. Scores were assigned to each pen and averaged across all pens in that treatment.

^2^Scale of 0 to 4, with 0 = no visible lesions; 1 = superficial lesions, points and lines; 2 = deeper skin lesion of small size (maximum size equal to tail diameter at respective location); 3 = deeper skin lesion of larger size (larger than tail diameter at respective location); and 4 = loss of tail length due to biting. Scores were assigned to each pen and averaged across all pens in that treatment.

### Carcass composition

Carcass composition assessed in each group of pigs marketed (day 14, 28, or 42) is presented in Table 4. At day 14, hot carcass weight was reduced in pigs fed the CaCl_2_ (*P* < 0.001) diet compared with CON, which averaged 112.4 kg. Percent lean and backfat tended to differ among dietary treatments at this time (*P* = 0.088 and *P* = 0.060, respectively), but no treatment groups significantly differed from another. Loin depth differed among treatments (*P* < 0.001), with pigs fed the CaCl_2_ diet tending to have reduced loin depth compared with CON (*P* = 0.094).

**Table 4.** Carcass composition after 14, 28, or 42 d in pigs fed diets intended to slow growth

Item	CON	CaCl_2_	Corn	LowIle	15% NDF	20% NDF	SEM	*P*-value
First harvest group (day 14)^1^								
HCW, kg	112.4^ab^	98.7^c^	108.8^ab^	115.0^a^	113.6^ab^	107.6^b^	2.05	<0.001
Lean, %	53.2	54.0	53.1	53.2	53.8	54.7	0.46	0.088
Backfat, mm	17.5	15.0	17.2	17.1	16.3	14.6	0.82	0.060
Loin depth, mm	65.6^ab^	61.2^b^	63.3^ab^	66.0^ab^	67.9^a^	67.6^a^	1.25	<0.001
Second harvest group (day 28) ^2^								
HCW, kg	117.1^a^	97.6^d^	107.0^c^	116.1^a^	115.6^ab^	110.8^bc^	1.46	<0.001
Lean, %	53.3^cd^	55.3^a^	52.6^d^	53.6^bcd^	53.8^bc^	54.5^ab^	0.28	<0.001
Backfat, mm	17.1^a^	13.1^c^	18.0^a^	16.9^a^	16.3^ab^	14.3^bc^	0.61	<0.001
Loin depth, mm	67.2^ab^	63.8^bc^	61.3^c^	68.4^a^	68.2^a^	67.9^a^	0.96	<0.001
Third harvest group (day 42) ^3^								
HCW, kg	114.9^a^	91.1^d^	103.6^c^	115.1^a^	114.1^a^	108.6^b^	1.67	<0.001
Lean, %	53.7^b^	55.5^a^	52.0^c^	54.2^b^	54.1^b^	54.7^ab^	0.30	<0.001
Backfat, mm	16.7^b^	12.7^d^	19.3^a^	15.7^bc^	15.8^bc^	14.5^cd^	0.54	<0.001
Loin depth, mm	68.7^a^	60.7^b^	59.3^b^	69.4^a^	68.9^a^	67.8^a^	1.00	<0.001

^1^ *n* = 8 pen/trt, 3 pigs/pen.

^2^ *n* = 8 pen/trt, 6 pigs/pen.

^3^ *n* = 8 pen/trt, 10 pigs/pen.

^a–d^Means within a row with differing superscripts differ significantly at *P* < 0.05.

At day 28 (second harvest group), the average hot carcass weight of pigs fed the CON diet was 117.1 kg. Hot carcass weight was reduced in pigs fed 20% NDF (*P* = 0.008), Corn (*P* < 0.001), and CaCl_2_ (*P* < 0.001) diets compared with CON. Percent lean was greater in pigs fed 20% NDF (*P* = 0.021) and CaCl_2_ (*P* < 0.001) diets compared with CON. Conversely, backfat depth was reduced in pigs fed 20% NDF (*P* = 0.022) and CaCl_2_ (*P* < 0.001) diets compared with CON. Loin depth was reduced in pigs fed the Corn (*P* = 0.001) diet compared with CON.

At day 42 (third harvest group), hot carcass weight was reduced in pigs fed 20% NDF (*P* < 0.001), Corn (*P* < 0.001), and CaCl_2_ (*P* < 0.001) diets compared with CON, which averaged 114.9 kg. Percent lean was reduced in pigs fed the Corn (*P* < 0.001) diet compared with CON, and increased in pigs fed the CaCl_2_ (*P* < 0.001) diet compared with CON. Backfat depth was increased in pigs fed the Corn (*P* = 0.002) diet compared with CON. Conversely, backfat depth was reduced in pigs fed 20% NDF (*P* = 0.011) and CaCl_2_ (*P* < 0.001) diets compared with CON. Finally, loin depth was reduced in pigs fed CaCl_2_ (*P* < 0.001) and Corn (*P* < 0.001) diets compared with CON.

### Pork loin quality

Pork loin quality traits were collected on a subset of pigs harvested at day 28 and were measured after 7 d of storage (Table 5). When expressed as a percentage of hot carcass weight, trimmed weight of a single loin did not differ among treatments (*P* = 0.115). Ultimate loin pH did differ (*P* = 0.015), with pigs fed the CaCl_2_ diet having reduced loin pH compared with CON (Contrast *P* = 0.022). Drip loss differed among treatments (*P* = 0.014); this was driven by an increase in drip loss in pigs fed CaCl_2_ and 20% NDF diets compared with those fed the Corn diet rather than any differences from CON. Cook loss did not differ among treatments (*P* = 0.457). Star probe measurements were greater in loin chops from pigs fed the CaCl_2_ diet compared with those of pigs fed the CON diet (*P* < 0.001) and all other dietary treatment groups. Hunter L color values differed overall (*P* = 0.016), which was driven by a tendency for pigs fed the Corn diet to have greater Hunter L values compared with CON (Contrast *P* = 0.078). Redness (Hunter a) did not differ among treatments. Yellowness (Hunter b) did differ (*P* = 0.045), driven by a tendency for pigs fed the Corn diet to have greater yellowness values compared with CON (Contrast *P* = 0.068). Color scores and firmness scores did not differ among treatments. Marbling did differ among treatments (*P* = 0.013), though no treatments differed from CON. However, pigs fed CaCl_2_ and 20% NDF diets had lesser marbling compared with those fed the Corn diet (*P* < 0.05 for both contrasts).

**Table 5.** Pork loin quality assessment after 28 d in pigs fed diets intended to slow growth. Loins were aged 7 days prior to analysis

Item	CON	CaCl_2_	Corn	LowIle	15% NDF	20% NDF	SEM	*P*-value
Trimmed loin weight, % of HCW^1^	4.06	4.26	4.03	4.27	4.23	4.24	0.078	0.115
pH	5.62^a^	5.53^b^	5.57^ab^	5.61^a^	5.57^ab^	5.55^ab^	0.020	0.015
Drip loss, %^2^	2.12^ab^	2.62^a^	1.72^b^	2.28^ab^	2.38^ab^	2.80^a^	0.211	0.014
Cook loss, %	19.0	19.1	18.5	18.2	20.0	19.0	0.620	0.457
Star probe, kg	4.88^b^	6.42^a^	5.05^b^	5.11^b^	5.47^b^	5.32^b^	0.205	<0.001
L	48.0^x^	48.1^x^	49.7^y^	48.1^x^	49.5^x^	49.1^x^	0.455	0.016
a	14.5	14.3	14.6	14.3	13.9	14.5	0.185	0.160
b	4.86^x^	4.89^x^	5.41^y^	4.90^x^	4.90^x^	5.10^x^	0.142	0.045
Color^3^	3.2	3.1	2.7	3.3	2.8	2.8	0.19	0.112
Firmness^4^	2.2	1.9	1.9	2.1	1.9	1.8	0.10	0.125
Marbling^5^	1.7^ab^	1.5^b^	2.0^a^	1.8^ab^	1.7^ab^	1.3^b^	0.13	0.013

^1^Weight of one loin, standardized to hot carcass weight.

^2^Weight lost after 24 hr in retail storage.

^3^Scale of 1 to 6 where 1 = pale pinkish-grey to white and 6 = dark purplish red.

^4^Scale of 1 to 3 where 1 = very soft and 3 = very firm.

^5^Scale of 1 to 10 where 1 = 1.0% intramuscular fat and 10 = 10.0% intramuscular fat.

^a–d^Means within a row with differing superscripts differ significantly at *P* < 0.05.

^x,y^Means within a row with differing superscripts tend to differ at *P* < 0.10.

## Discussion

Market disruptions preventing the movement of market-ready pigs to slaughter facilities presents a unique challenge for pork producers. A variety of options to slow pig growth rates can be implemented at any stage of pig growth, including in pigs that are already near or at market body weights. However, it is challenging to slow or arrest the growth of late finishing pigs. Further, the late finishing period is a critical timepoint for fat deposition, and concerns regarding carcass composition and quality with dietary manipulation are particularly warranted. Thus, this study aimed to evaluate several dietary strategies that could be implemented to slow the growth of pigs in the late finishing period, as well as evaluate effects on carcass composition, pork loin quality, and behavioral vices.

Decreasing the dietary electrolyte balance (**dEB**) or dietary undetermined anion of a pig diet via the addition of an anion such as chloride as ammonium chloride or calcium chloride is one technique that can be implemented to suppress feed intake (Yen et al., 1981). Indeed, pigs fed the CaCl_2_ diet in the current study had the most considerable reductions in growth and feed intake. It is proposed that calcium chloride suppresses appetite through induction of metabolic acidosis, as increasing the plasma chloride level subsequently creates a plasma bicarbonate deficiency (Yen et al., 1981). In growing gilts, Pluske et al. (2015) observed 4% calcium chloride + 2.2% sodium tripolyphosphate in the diet reduced feed intake by 30% in the first week, but this amount was halved for the remainder of the experiment. In subsequent weeks, feed intake and growth continued to be reduced to a lesser degree, for an overall reduction in ADG of 15% over the 21 d study (Pluske et al., 2015). Other researchers have also observed reductions in growth and feed intake with the use of calcium chloride in the diet (Yen et al., 1981; Patience et al., 1987; Haydon et al., 1990; Dersjant-Li et al., 2001), albeit the extent of this reduction varies based on the inclusion the rate of calcium chloride, the addition of other salts, and age of the pig. Although feeding late finisher pigs an acidogenic diet may provide the most considerable reductions in growth rates, very little information regarding the influence of acidogenic diets on carcass composition and fresh pork quality is available.

In the current study, pigs fed the CaCl_2_ diet had lighter, leaner carcasses regardless of how long pigs were fed this diet. This is partially attributed to the size of these pigs at the time of harvest compared with pigs fed the CON diet, as loin weight did not differ when expressed as a percentage of carcass weight. Irrespective of these changes to carcass composition, alterations to fresh pork quality are potentially a concern with feeding an acidogenic diet. Inducing metabolic acidosis reduces muscle pH, facilitating the development of PSE meat. However, Dugan et al. (1997) reported that postmortem infusion of calcium chloride and Ethylene glycol-bis (2-aminoethylether)-*N*,*N*,*N*′,*N*′-tetraacetic acid, while inducing a PSE-like condition exemplified by rapid pH decline and greater drip loss, resulted in tenderization of semimembranosus muscle. However, this study involved postmortem infusion of calcium chloride, and it is unlikely dietary supplementation would result in similar increases to the amount of free calcium in the muscle (Dugan et al., 1997). With regards to dietary manipulation, the addition of ammonium chloride 5 d prior to slaughter to reduce dEB did not significantly affect color (L, a, b), water holding capacity, or 24 hr muscle pH (Ahn et al., 1992). Although these treatments were implemented for only 5 d preslaughter, metabolic acidosis was apparent when measured via blood parameters (Ahn et al., 1992). Similarly, pigs fed a mineral salt diet containing 3% calcium chloride dihydrate and 1.6% sodium tripolyphosphate for 28 d prior to slaughter resulted in greater 45 min pH, but no difference in ultimate pH or other pork quality parameters (Moore et al., 2016). However, Moore et al. (2016) reported no growth performance or feed intake reductions as a result of this dietary treatment, possibly due to differences in the types of salts added, which makes it difficult to compare to studies in which performance reductions are noted.

In the current study, pigs fed a 3% calcium chloride diet had a reduction in loin pH compared with loins from pigs fed the control diet after 7 d of storage. This was accompanied by a greater star probe value than loins from all other diets. Greater star probe values are associated with reduced sensory tenderness (Huff-Lonergan et al., 2002; Schulte et al., 2019), indicating reduced loin tenderness in pigs fed calcium chloride. The increased star probe observed herein is potentially a function of lowered ultimate pH, which is known negatively influence a variety of pork quality metrics (Lonergan et al., 2007; Schulte et al., 2019). Reduced pH and tenderness suggest the potential for undesirable impacts on eating quality, particularly at a higher inclusion rate of calcium chloride or if diets were provided throughout the growing period. However, given the highly significant reductions in growth (94%) and feed intake (43%) observed at a 3% inclusion rate, feeding a greater amount, or for a longer duration, may not be practical. Regardless, if the objective is to stop growth rate due to impaired access to a harvest facility, feeding 3% calcium chloride was the most effective strategy as it was accompanied by near cessation of growth.

Manipulations to dietary amino acids represent a cost-effective option to slow the growth rates of finishing pigs, which limit capacity for skeletal muscle protein synthesis and lean accretion. In the current study, 2 amino acid deficiency strategies were studied. One strategy examined overall amino acid deficiency via eliminating soybean meal and synthetic amino acids, thus including only ground corn and vitamins and minerals (Corn diet). In contrast, the other strategy examined a low isoleucine diet formulation (LowIle diet). Limiting protein and many essential amino acids is documented to slow growth rates of both finishing pigs and grower pigs (Easter and Baker, 1980; Adeola and Young, 1989; Cromwell et al., 1993; Kerr et al., 1995; Ruusunen et al., 2007). In agreement with these reports, feeding a diet deficient in many essential amino acids to late finishing pigs resulted in attenuated body weight gains compared with those fed diets meeting or exceeding requirements. However, it is important to note that the Corn diet did not affect feed intake. Limiting protein deposition may repartition energy towards being deposited as fat, and thus induce a shift in body composition. Indeed, when pigs are deficient in protein throughout the grower period, a reduction in lean tissue and increase in backfat has been observed (Easter and Baker, 1980; Kerr et al., 1995; Ruusunen et al., 2007; Morazán et al., 2015). Similarly, feeding low dietary protein for a period of 21 to 35 d can substantially increase the intramuscular fat in finisher pigs (Cisneros et al., 2010) and feeding high amounts of corn to create protein deficiency throughout the growing period results in pigs having greater loin marbling and lighter color compared with those fed a diet adequate in protein (Kerr et al., 1995). In the current study, a greater amount of backfat and lesser percentage of lean tissue was observed after these pigs had been fed the Corn diet for 42 d, despite producing a smaller carcass. However, no changes were observed in marbling scores, suggesting that greater fat accretion did not result in greater intramuscular fat deposition, possibly due to the short duration of time (14 to 42 d) in which these diets were fed. Regardless, despite the reduction in lean percentage and increased backfat that accompanied this diet, total amino acid restriction still provided significant reductions in growth rates, representing a cost-effective and easily implemented strategy for pork producers required to slow pig growth.

Similar to reducing all amino acids, reductions in growth may be achieved through the reductions of specific amino acids. Particularly, creating a deficiency in a singular branched chain amino acid (**BCAA**), such as isoleucine, acts to both limit skeletal muscle protein synthesis as well as potentially feed intake. It is thought that by reducing or increasing a singular BCAA, the balance among all BCAAs is disrupted, which interact antagonistically when not balanced properly (Harper et al., 1984). Pigs fed 0.25% to 0.28% isoleucine in the late finisher phase had depressed growth performance and feed intake when compared with those fed adequate levels (0.55%) of isoleucine (Parr et al., 2004). Similarly, isoleucine deficiencies suppress the feed intake and growth rate of nursery pigs (Kerr et al., 2004). Extreme isoleucine deficiency mobilizes adipose tissue and suppresses lipogenesis (Du et al., 2012); however, moderate isoleucine reductions may preferentially suppress skeletal muscle synthesis (Zhang et al., 2016). In nursery pigs, reduced dietary isoleucine is associated with reduced abundance of glucose transporters in the skeletal muscle (Zhang et al., 2016), suggesting that isoleucine deficiency disproportionately reduces skeletal muscle energy uptake and synthesis of skeletal muscle tissue, which may lead to excess energy consumed by the pig being deposited into fat stores. However, in the current study, feeding a diet deficient in SID isoleucine (0.33%) did not appear to modulate growth rates, feed intake, or carcass composition.

Finally, growth rate reduction could be achieved by reducing the energy density and increasing the fiber content of the diet. In general, this can be done by the inclusion of high levels of ingredients such as distillers dried grains with solubles, soybean hulls, or wheat middlings, depending on ingredient availability and price. Fibrous ingredients increase digesta volume and allow for satiety while providing a lower amount of energy compared with a less fibrous diet (Kerr and Shurson, 2013). In the current study, soybean hulls were used to achieve either a moderate or high dietary NDF of 15% and 20%, respectively. Previous researchers have reported that feeding high fiber diets slows growth rates and may or may not reduce feed intake depending on the fiber type and inclusion rate (Powley et al., 1981; Varel et al., 1984; Pond et al., 1988; Déru et al., 2020). In the current study, a dietary NDF of 20%, but not 15%, reduced both growth and feed intake of late finishing pigs, suggesting that an extremely high dietary fiber level is required to suppress energy intake. Feeding the 20% NDF diet reduced carcass weight of pigs marketed compared with those fed a standard diet as a result of slower growth rates, similar to what has been observed previously (Arkfeld et al., 2015). Further, pigs fed high fiber diets throughout the grower period have reduced backfat and greater lean percentage compared with those fed a lower fiber diet (Arkfeld et al., 2015; Déru et al., 2020). This may depend on whether or not diets are isocaloric, as when energy is held constant, pigs fed high fiber diets have lesser dressing percentage, reduced loin depth, and no change to amount of backfat compared with those fed a lower fiber diet (Weber et al., 2015). Results of the current study, in which diets were not isocaloric, demonstrate pigs fed 20% NDF diets have reduced backfat after 28 or 42 d on dietary treatments. Limited changes to percent fat free lean were observed, possibly due to the short amount of time these pigs were fed high fiber diets compared with the study by Arkfeld et al. (2015). The current study also reported no differences in pork loin quality metrics due to feeding high fiber diets. Previously, Arkfeld et al. (2015) showed some changes to pork color in pigs fed high fiber diets (~26% NDF), with reduced lightness (L*) but no changes to a* or b* values compared with pigs fed 9% NDF diets. Conversely, Déru et al. (2020) observed no differences in L* values but reductions in both a* and b* values. However, both studies continuously fed high-fiber diets throughout the growing period, suggesting that differences in meat color may be observed when high-fiber diets are fed for a long duration of time, but unlikely to be observed when fed for a shorter period of time.

Often, restricting growth and feed intake in group housed pigs leads to an increase in behavioral vices such as ear and tail biting, presenting a major welfare issue (Smulders et al., 2008). An increase in abnormal behavioral problems can be associated with a diet that does not provide sufficient satiety (De Leeuw et al., 2008) or when animals are limit-fed, such as breeding sows (Meunier-Salaun et al., 2001). Thus, evidence of behavioral vices was assessed weekly. No evidence of increased ear and tail biting was observed among any of the dietary treatments, suggesting that all these diets, when fed to late finishing pigs for this period of time, do not result in social vices. This may be due to the fact that pigs achieved satiety on all of the dietary treatments evaluated herein.

In the case of market disruptions that necessitate slowing the growth of finishing pigs, pork producers need reliable and economical dietary manipulations that come with the least negative impacts on carcass composition, pork quality, and behavioral vices. The results of this study provide pork producers several viable dietary strategies that could be implemented to slow growth of commercially housed late finishing pigs, particularly 3% CaCl_2_, 97% corn, and 20% NDF. None of the diets measurably increased behavioral vices, quelling a major welfare concern. Granted, alternative genetic lines could respond differently both with respect to behavioral vices and growth performance, so producers must monitor and validate the observations of this study if and when implemented. Although producing a high-quality carcass is likely not the primary goal of producers needing to implement growth reducing strategies, it is important to note that reductions in growth were accompanied by changes to carcass composition and pork quality. Particularly, both CaCl_2_ and 20% NDF diets tended to produce a smaller, leaner carcass, whereas feeding 97% corn resulted in a greater amount of fat deposition at the expense of lean muscle tissue. Further, the reduced ultimate pH and higher star probe values in loins from pigs fed the CaCl_2_et suggest the potential for reduced eating quality. Although none the observed changes to carcass composition and quality were large, it is possible quality parameters would be more negatively affected if pigs were fed dietary treatments for longer than 42 d. Regardless, if stopping growth of late finishing pigs is necessary, CaCl_2,_ 97% corn, and 20% NDF all accomplish this objective without significant reductions to eating quality.

## Abbreviations

ADFI: average daily feed intake
ADG: average daily gain
BCAA: branched-chain amino acid
BW: body weight
CaCl_2_: calcium chloride
dEB: dietary electrolyte balance
G:F: gain:feed
Ile: isoleucine
NDF: neutral detergent fiber
PSE: pale, soft, exudative
SID: standardized ileal digestible
